# Pseudocyst and a Collar Stud Abscess: New Face of Necrotizing Enterocolitis

**Published:** 2014-10-20

**Authors:** Hemant Kumar, Ramnik Patel, Nitin Patwardhan, Bharat More

**Affiliations:** Department of Paediatric Surgery, Directorate of Children’s Services, Univeristy Hospitals of Leicester NHS Trust, Leicester Royal Infirmary , Infirmary Square, Leicester LE1 5WW United Kingdom

**Keywords:** Necrotizing enterocolitis, Pseudocyst, Collar stud abscess, Intestinal perforation, Preterm

## Abstract

Meconium pseudocyst formation secondary to antenatal perforation is well described. We present a preterm infant who had intra-abdominal pseudocyst formation following postnatal intestinal perforation secondary to necrotizing enterocolitis (NEC) and secondarily leading to extra-abdominal collar stud abscess. This is new face of NEC and this presentation has not been reported earlier.

## INTRODUCTION

Despite advances in neonatal care, NEC still remains as one of the most common causes of surgical intervention in the neonate [1]. Abdominal radiography is an important component of the assessment of infants with suspected NEC. Rarely, localised pneumoperitoneum secondary to intestinal perforation could be missed [2]. 

We present a neonate who had medical treatment for NEC initially and later developed silent perforation, which subsequently got walled off in the form of pseudocyst. This led to collar-stud abscess of the anterior abdominal wall, with pus and free air tracking under the abdominal skin. To the best of our knowledge, such pseudocysts associated with collar-stud abscesses have not hitherto been reported.

## CASE REPORT

A male neonate weighing 730 gram was born at 24+2/40 week gestation. Antenatal scans did not reveal any bowel abnormality. He was seriously ill at birth. The APGAR was 2 at 1 minute, and he was intubated at 10 minutes of age, surfactant (CurosurfR) was administered at 20 minutes and repeated at 12 hours. He required high frequency ventilation from day 13 to day 15 for worsening respiratory acidosis and increased oxygen requirement on conventional ventilation. 

Gut priming was commenced on day 2. Enteral feeds were poorly tolerated with frequent bilious aspirates. Feeds were increased slowly, however full feeds were not established. PDA was noted and required inotropic support. In view of the bilious aspirates along with abdominal distension, the neonate was commenced on intravenous antibiotics for suspected sepsis/NEC.

Serial abdominal radiographs in the second week showed an abnormal extra luminal gas shadow in right lower quadrant and a gradually increasing soft fluctuant swelling just above the gas shadow on lateral decubitus view (Fig. 1). Surgical opinion was sought in view of abnormal localised extra intestinal gas shadow in the same position seen on repeated abdominal radiographs. No evidence of pneumatosis intestinalis was noted in abdominal radiographs. 

**Figure:**
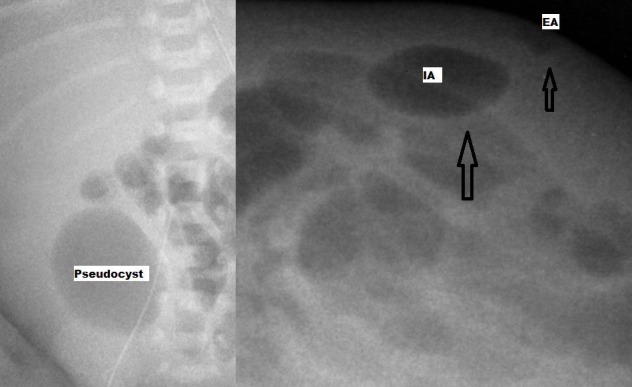
Figure 1: Initial AXR with loculated free gas in right lower abdomen and decubitus showing dumbbell swelling in lower abdomen.

He was continued to be unwell on ventilator with inotropic support, and blood products transfusions. On day 14, he developed an extra-abdominal soft fluctuant swelling in the right lower quadrant of the abdomen suggestive of an extra abdominal component of the collar stud abscess. 

A repeat abdominal and lateral tangential view showed a large pocket of loculated extra-intestinal air in the right iliac fossa with another air pocket in the subcutaneous tissue (Fig. 2). Based on our previous experience and with worsening clinical, laboratory and radiological findings; an intra-abdominal walled off perforation with pseudocyst formation in association with an extra-abdominal pocket of collar stud abscess was suspected.

**Figure:**
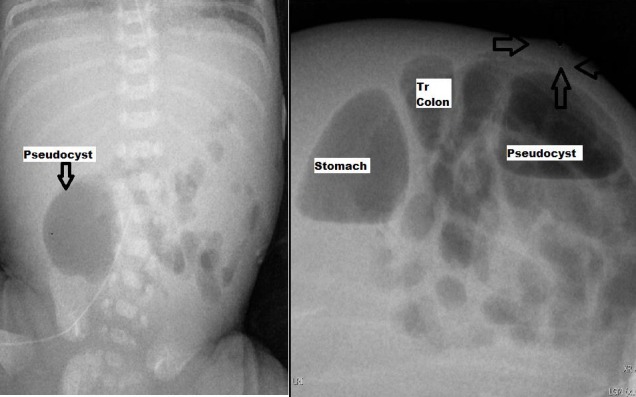
Figure 2: Delayed Abdominal film showing persistence of air pocket and decubitus showing air in collar stud abscess.

He underwent laparotomy, which revealed an ileo-caecal segmental NEC with perforation and pseudo cyst formation and a defect in the abdominal wall leading to abscess cavity in the subcutaneous tissue. The pseudocyst contained air and intestinal contents. No intestinal atresias or other bowel pathology were noted. 

The infant underwent right hemicolectomy and stoma formation at the site of collar stud abscess after drainage and debridement. The healthy proximal small bowel measured 75cm. 

He was tested for cystic fibrosis, and the genetic test was negative. He was established on feed subsequently and stoma was reversed in 6 week time, he has quickly re-established on feed, and at discharge was feeding on demand. At 18 month follow up he was thriving well.

## DISCUSSION

The recognized late complications of necrotizing enterocolitis are intestinal stenosis, internal fistulae, malabsorption and an intervening enterocyst between extensive proximal and distal colonic strictures [3-5]. Enterocyst formation has been reported as late complication of NEC; hitherto there is no report on acute phase postnatal pseudocyst. Recently, pseudocyst following neonatal appendicitis has been reported in a term neonate simulating an intestinal or duodenal duplication cyst but none in the preterm infants in the NEC [6].

Meconium peritonitis is a sterile chemical peritonitis caused by antenatal bowel perforation with intraperitoneal extravasations of the meconium in utero. Meconium pseudocysts are usually associated with bowel obstruction secondary to atresias or cystic fibrosis; our infant had no atresias or other pathology. Raised inflammatory markers, abnormal abdominal radiographs and presence of feed residue in the cysts clearly do not suggest an antenatal event.

The deterioration may be manifested by increasing abdominal distension and tenderness, abdominal wall erythema /and or persistent acidosis, oliguria and cardio-vascular instability. With the availability of newer broad-spectrum antimicrobials, and better supportive measures, the infection is contained, and the neonates run a protracted course, with the development of the pseudocyst. 

A fixed loculated abnormal extra luminal gas shadow may be visible on serial abdominal radiographs. The free air and the pus can then track and form a collar stud abscess, which was evident in our case; clinical, laboratory, radiological and operative findings supported these observations. An abdominal mass or abscess with persistent sepsis and worsening clinical features requires surgery [7].

The development of this new presentation of the classic NEC could be due to several reasons. The neonatal team in established cases of NEC prefers straight institution of higher antibiotics rather than staged approach of traditional triple 1st generation antibiotics particularly in very premature and less than 1 Kg birth weight babies. This slows down the rapid progression of the disease and allows time for omental walling off of the diseased segments of bowel. Even if it leads to perforation, it will be limited and walled off perforation rather than free perforation and frank pneumoperitoneum as the baby has bought some more time to build the immune response and higher antibiotics helps in curbing the extent and severity off the disease as well [8-10]. 

The greater omentum allows for its pockets to be formed and if carefully interpreted it may be possible to find free gas signs [8-10]. Our cases had signs of free gas pockets in the abdominal and decubitus films retrospectively [7, 10].

The development of the collar stud abscess seems to follow the natural neurovascular foramina to get the extension in the subcutaneous plane with an intervening bottleneck of the abdominal wall muscles and fascial layers. Early recognition and appropriate surgical intervention prevents morbidity and mortality.

## Footnotes

**Source of Support:** Nil

**Conflict of Interest:** None

